# Facile self-assembly of colloidal diamond from tetrahedral patchy particles via ring selection

**DOI:** 10.1073/pnas.2109776118

**Published:** 2021-11-24

**Authors:** Andreas Neophytou, Dwaipayan Chakrabarti, Francesco Sciortino

**Affiliations:** ^a^School of Chemistry, University of Birmingham, Edgbaston, Birmingham B15 2TT, United Kingdom;; ^b^Dipartimento di Fisica, Sapienza Università di Roma, 00185 Roma, Italy

**Keywords:** colloidal self-assembly, tetrahedral networks, tetrahedral patchy particles, diamond lattice, polytype selection

## Abstract

Diamond-structured crystals, particularly those with cubic symmetry, have long been attractive targets for the programmed self-assembly of colloidal particles, due to their applications as photonic crystals that can control the flow of visible light. While spherical particles decorated with four patches in a tetrahedral arrangement—tetrahedral patchy particles—should be an ideal building block for this endeavor, their self-assembly into colloidal diamond has proved elusive. The kinetics of self-assembly pose a major challenge, with competition from an amorphous glassy phase, as well as clathrate crystals, leaving a narrow widow of patch widths where tetrahedral patchy particles can self-assemble into diamond crystals. Here we demonstrate that a two-component system of tetrahedral patchy particles, where bonding is allowed only between particles of different types to select even-member rings, undergoes crystallization into diamond crystals over a significantly wider range of patch widths conducive for experimental fabrication. We show that the crystallization in the two-component system is both thermodynamically and kinetically enhanced, as compared to the one-component system. Although our bottom-up route does not lead to the selection of the cubic polytype exclusively, we find that the cubicity of the self-assembled crystals increases with increasing patch width. Our designer system not only promises a scalable bottom-up route for colloidal diamond but also offers fundamental insight into crystallization into open lattices.

## Significance

The self-assembly of colloidal diamond–a classic example of an open crystal with the low coordination number of four and much sought after due to its applications in visible light management–from designer spherical colloidal particles has proved challenging over the years. The formation of the diamond lattice from tetrahedral patchy particles is hampered by the propensity to form competing open periodic structures for narrow patches or dynamically arrested states for wider patches, leaving a narrow window in design space where diamond crystals may be realized. Our two-component system of designer tetrahedral patchy particles supports a significantly wider range for patch sizes for programmed self-assembly, thus facilitating experimental fabrication, and offers fundamental insight into crystallization into open lattices.

Much effort has been placed in devising fabrication routes to diamond-structured colloidal crystals, driven by their applications in visible photonics (1, 2). In this context, the self-assembly of submicrometer colloidal particles has long been recognized as a promising scalable bottom-up approach (3–6). In this endeavor, a variety of designer building blocks with tunable interparticle interactions have been synthesized over the years, leading to recent success in their self-assembly into diamond-structured crystals (7, 8). Although spherical particles decorated with four patches in tetrahedral symmetry appeared as the front-runner to yield a diamond crystal (9, 10), the self-assembly of a diamond crystal from such tetrahedral patchy particles proved challenging.

The self-assembly of a diamond crystal from tetrahedral patchy particles faces challenges that can be of both thermodynamic and kinetic origins, as suggested by a number of computer simulation studies (11–14). When the particles possess narrow patches, five-member rings form more readily as compared to six-member rings, and clathrate structures are thus kinetically favored over a diamond structure (14). On the other hand, when patches are too wide, there is not a sufficiently large thermodynamic driving force for spontaneous crystallization to occur (11). A significant degree of supercooling is then required for the free-energy barrier to crystal nucleation to become surmountable to allow for spontaneous crystallization (12). As a result, crystallization is preempted by the dynamic arrest of the system into an amorphous glassy network when the attractive patches are too wide (11, 12, 14). The glassy network has a distribution of rings, including even- and odd-member rings (15). As diamond crystals comprise exclusively even-member rings, the tetrahedral patchy particles become dynamically arrested due to frustration between the local order of the fluid and the global order of the crystal (16).

Recent studies have demonstrated that a two-stage self-assembly scheme for triblock patchy particles via tetrahedral clusters promotes crystallization into open crystals, where each particle has a coordination number of six, by suppressing the formation of five- and seven-member rings (17, 18). The frustration caused by a distribution of ring sizes could be a generic mechanism by which crystallization into an open lattice is hindered. In the present computational study, we sought to demonstrate the generality of this hypothesis by investigating the self-assembly of diamond crystals in a system that contains two types of designer tetrahedral patchy particles, each having four identical patches, in a 1:1 mixture. We introduce specific interactions, such that bond formation can only occur between the two different types of particles, labeled A and B. Such specificity in bond formation can be realized using DNA-mediated interactions (19–21). Our tetrahedral patchy particles are represented by the widely used Kern–Frenkel model (22), where particles of different types interact via a combination of a hard-core repulsion, with diameter *σ*, and a square-well attraction modulated by an angular factor between the patches, with a half-angle *θ*. The angular factor is unity only when the patches are properly oriented, that is, the vector connecting the centers of the two particles passes through the patches on their surfaces, and zero otherwise. The width of the square well, *δ*, determines the range of the attraction between the patches relative to the particle diameter and is set to δ=0.2σ. The depth of the square well, *ε*, governs the strength of the bond. Only hard-sphere repulsion is at play between particles of the same type.

**Fig. 1.**
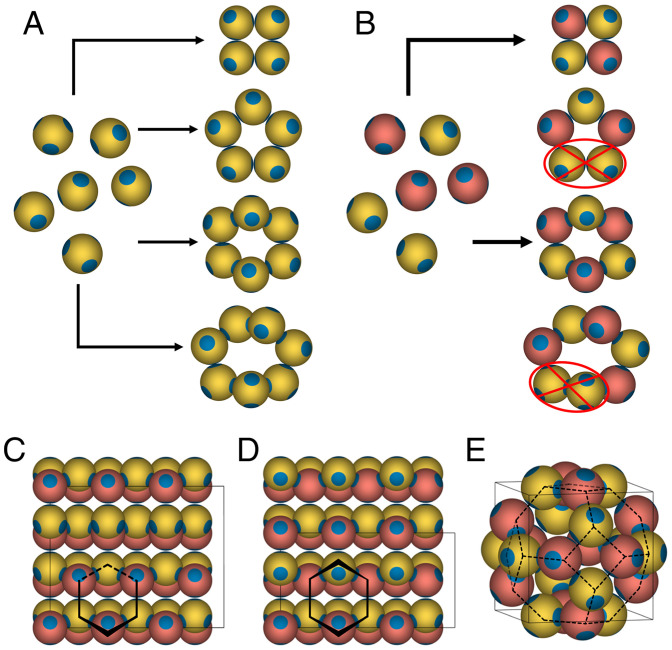
Design strategy for the selection of even-member rings in a system of tetrahedral patchy particles using specific interactions. (*A*) Examples of four-, five-, six-, and seven-member rings that can form in a one-component system of tetrahedral patchy particles. (*B*) Examples four- and six-member rings that can form in a two-component system of tetrahedral patchy particles, where bonds can form only between distinct species, labeled A (colored yellow) and B (colored pink). Examples of five- and seven-member rings are also shown to highlight that odd-member rings cannot form in such a two-component system of tetrahedral patchy particles, as they require A–A or B–B bonds. (*C–E*) Example crystal structures that can be stabilized by a two-component system of tetrahedral patchy particles: (*C*) cubic diamond, (*D*) hexagonal diamond, and (*E*) clathrate s-III. The thin black lines represent the edges of the respective unit cells of the crystal structures. We highlight representative six-member rings present in the cubic and hexagonal diamond crystals in the chair and boat conformations, respectively. Additionally, we show the underlying network in the clathrate s-III structure with dashed lines to highlight that the four- and six-member rings in the unit cell are arranged such that they form a truncated octahedron.

Chains of particles in the two-component system considered here can only form closed bond loops in the cases where the first and last particles in the chain are of different species. The contrasting scenarios between the formation of rings in a one-component and a two-component system of tetrahedral patchy particles are shown in Fig. 1 *A* and *B*, vividly demonstrating the selection of even-member rings imposed by the constraint of interspecies binding only. The two-component system of tetrahedral patchy particles can, of course, form cubic and hexagonal diamond crystals, representative views of which are shown in Fig. 1 *C* and *D*, respectively, and in *SI Appendix*, Fig. S1. Additionally, we recognize that the two-component system of tetrahedral patchy particles should effectively suppress the formation of s-I and s-II clathrates (both lattices requiring odd-member rings), but it can still stabilize the s-III clathrate, which is composed of four- and six-member rings, as shown in Fig. 1*D* (23). However, due to the presence of four-member rings, this structure can only form when $\theta \geq 9.75^{{}^{\circ}}$; additionally, the six-member rings found in this structure are planar, as compared to the boat and chair conformations found in the diamond crystals (as shown in Fig. 1 *C* and *D*) (11). Due to the frustration associated with the formation of four- and planar six-member rings, we anticipate our design rules for diamond will not suffer any competition from the clathrate s-III crystal.

## Results and Discussion

A two-component system of tetrahedral patchy particles with specificity of interactions to allow for only interspecies binding then appears as a propitious route to a diamond crystal. To explore whether this is indeed the case, we carried out Monte Carlo simulations of one-component and two-component systems of *N* = 1000 tetrahedral patchy particles in the canonical (*NVT*) ensemble. We used an equal number of A and B particles in the two-component system (i.e., $N_{\text{A}}=N_{\text{B}}$ = 500). Additionally, we considered systems where the patch half-angles for the tetrahedral patchy particles were chosen such that diamond was not expected to be kinetically accessible in the one-component system.

**Fig. 2.**
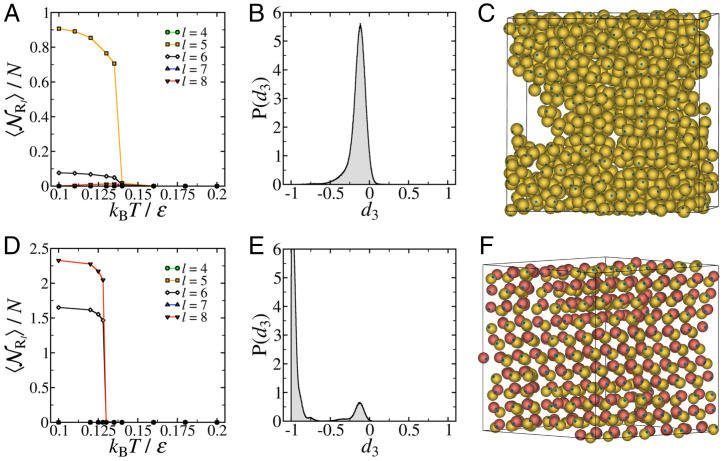
Comparison of the self-assembly in one- and two-component systems of tetrahedral patchy particles with narrow patches. Self-assembly in (*A–C*) one-component and (*D*–*F*) two-component systems of *N* = 1000 tetrahedral patchy particles with patch half-angle $\theta =12^{{}^{\circ}}$ at $\rho^{*}=N\sigma^{3}/V=0.3$. (*A* and *D*) Evolution of the average number of rings of length *l* ($N_{{\text{R}}_{l}}$) with temperature. (*B* and *E*) Probability distribution function of the translational order correlation parameter *d*_3_, defined in *Materials and Methods*, at a temperature of $k_{\text{B}}T/\varepsilon =0.1$. (*C* and *F*) Representative snapshots of the crystalline configurations in the two systems at a temperature of $k_{\text{B}}T/\varepsilon =0.1$.

In Fig. 2, we compare the self-assembly observed in a one-component (Fig. 2 *A–C*) and a two-component (Fig. 2 *D–F*) system of tetrahedral patchy particles with $\theta =12^{{}^{\circ}}$ at $\rho^{*}=N\sigma /V=0.3$. We find that both systems undergo a first-order phase transition upon gradual cooling; the one-component system forms a clathrate structure, while the two-component system forms a diamond structure instead. This is reflected in Fig. 2 *A* and *D*, where we compare the evolution of the average number of rings of length *l* ($N_{{\text{R}}_{l}}$) with temperature for the one-component and two-component systems, respectively. The first-order phase transition in the one-component system is marked by a dominant growth of five-member rings, as one would expect for a clathrate structure (14). In contrast, a sharp increase in six- and eight-member rings in the two-component system signals self-assembly into a diamond crystal. The characteristic peaks in the probability distribution for the translational order correlation parameter, *d*_3_ (defined in *Materials and Methods*), as evident in Fig. 2 *B* and *D*, confirm the respective crystal structures. Fig. 2 *C* and *F* shows representative snapshots of the clathrate and diamond structures self-assembled in the one-component and two-component systems, respectively.

The distribution of *d*_3_ is routinely used to identify the formation of diamond in systems of tetrahedral patchy particles, since a pure cubic diamond structure has a single peak at $d_{3}=-1$, while hexagonal diamond and also random stacking diamond have an additional peak centered at $d_{3}=-0.115$ (9, 11, 12, 24, 25). The peak at $d_{3}=-1$ arises due to the presence of bonded pairs of particles (i.e., two particles that share a patch–patch bond) with staggered patches, while the peak at $d_{3}=-0.115$ corresponds to bonded particles with eclipsed patches. Fig. 2*B* reveals that the one-component system contains exclusively eclipsed bonds–a characteristic feature of the clathrate structures (26). In contrast, Fig. 2*E* shows that the two-component system contains predominantly staggered bonds, with some eclipsed bonds, suggesting that a mixture of cubic and hexagonal diamond has formed. At higher densities, we find that the one-component system crystallizes into a diamond structure only 10% of the time, alongside forming an amorphous clathrate phase (14), as shown in *SI Appendix*, Fig. S2.

**Fig. 3.**
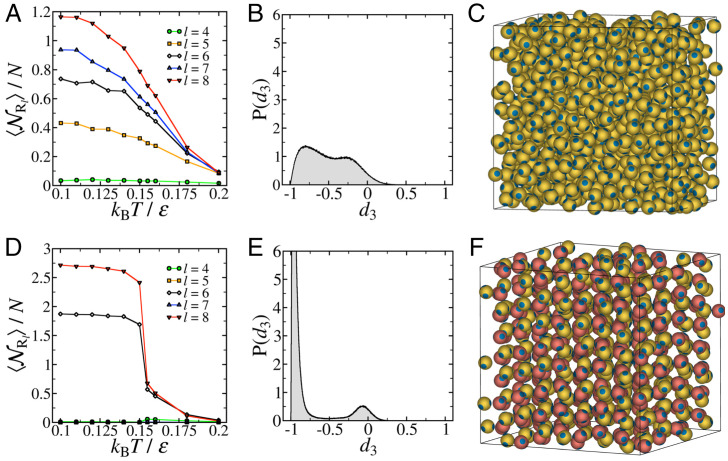
Comparison of the self-assembly in one- and two-component systems of tetrahedral patchy particles with wide patches. Self-assembly in (*A–C*) one-component and (*D*–*F*) two-component systems of *N* = 1000 tetrahedral patchy particles with patch half-angle $\theta =25^{{}^{\circ}}$ at $\rho^{*}=N\sigma^{3}/V=0.5$. (*A* and *D*) Evolution of the average number of rings of length *l* ($N_{{\text{R}}_{l}}$) with temperature. (*B* and *E*) Probability distribution function of the translational order correlation parameter *d*_3_ at a temperature of $k_{\text{B}}T/\varepsilon =0.1$. (*C* and *F*) Representative snapshots of the configurations in the two systems at a temperature of $k_{\text{B}}T/\varepsilon =0.1$.

Fig. 3 shows a comparison between the self-assembly of a one-component (Fig. 3 *A–C*) and a two-component (Fig. 3 *D–F*) system of tetrahedral patchy particles with a larger patch width having $\theta =25^{{}^{\circ}}$ at $\rho^{*}=0.5$. This *θ* value is well into the regime where a one-component system of tetrahedral patchy particles is known to become dynamically arrested into a glassy state upon cooling (11, 14). Fig. 3*A* shows that a distribution of rings with l∈[4−8] indeed emerges in the one-component system, with no signature of crystallization taking place, as revealed in Fig. 3*B* by the lack of well-resolved peaks in the distribution of *d*_3_. The broad distribution in *d*_3_ in this case suggests that there are a number of particles which form bonds that are neither staggered nor eclipsed. This implies that the resulting structure is truly amorphous, and not disordered because of polycrystallinity. In contrast, the two-component system readily crystallizes into a diamond structure, with the evolution of the number of rings with temperature, and the distribution of *d*_3_ at low temperatures, closely matching those of the two-component system with narrower patches, as evident in Fig. 3 *D* and *E*, respectively. Fig. 3 *C* and *F* shows representative snapshots of the configurations in the two systems at a low temperature, confirming the lack of crystalline order in the one-component system as opposed to the two-component system.

**Fig. 4.**
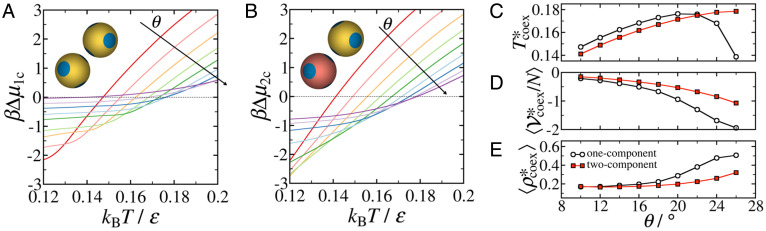
Thermodynamics of crystallization in one- and two-component systems of tetrahedral patchy particles as a function of patch width. Dependence of the chemical potential difference between the cubic diamond (cd) and fluid phases for (*A*) one-component ($\Delta \mu_{\text{1c}}$) and (*B*) two-component ($\Delta \mu_{\text{2c}}$) systems of *N* = 216 tetrahedral patchy particles on temperature ($k_{\text{B}}T/\varepsilon$) and patch half-angle (*θ*) at a pressure of $P^{*}=P\sigma^{3}/\varepsilon =0.03$. Here, $\Delta \mu =\mu_{\text{cd}}-\mu_{\text{fluid}},\;\beta ={\left(k_{\text{B}}T\right)}^{-1}$, and $\theta =10^{{}^{\circ}},12^{{}^{\circ}},14^{{}^{\circ}},16^{{}^{\circ}},18^{{}^{\circ}},20^{{}^{\circ}},22^{{}^{\circ}},24^{{}^{\circ}},26^{{}^{\circ}}$. The arrows show the direction of increasing *θ*. (*C–E*) Dependence of the coexistence temperature, $T_{\text{coex}}^{*}$, the fluid potential energy ($V_{\text{coex}}^{*}$), and the fluid density ($\rho_{\text{coex}}^{*}$) at the coexistence temperature on the patch half-angle *θ* for the one-component and two-component systems at $P^{*}=0.03$. The *θ*-axis label appears only for *E*.

We have thus established that our two-component system of designer tetrahedral patchy particles readily crystallizes into diamond crystals, even for patch half-angles where the diamond crystals are kinetically inaccessible for the one-component system. The facile crystallization of diamond for a two-component system of tetrahedral patchy particles can be predominantly attributed to the removal of kinetic traps containing odd-member rings from the energy landscape. However, in order to assess whether crystallization is also facilitated in the two-component system from thermodynamic considerations, we evaluate the thermodynamic driving force for crystallization (i.e., the chemical potential difference between the crystal and fluid phases: $\Delta \mu =\mu_{\text{crystal}}-\mu_{\text{fluid}}$). Fig. 4 *A* and *B* shows the temperature and *θ* dependence of Δμ for the one-component ($\Delta \mu_{\text{1c}}$) and two-component ($\Delta \mu_{\text{2c}}$) systems, respectively. At higher temperatures, where the fluid is more stable than the crystal, we see that $\Delta \mu_{\text{2c}}>\Delta \mu_{\text{1c}}$ for all *θ* values considered, implying that the two-component fluid phase is more stable than its one-component counterpart, relative to their respective crystalline phases. This additional stability can be mainly attributed to the entropy of mixing: $\Delta S_{\text{mix}}/(Nk_{\text{B}})=-\sum_{i=\text{A,B}}\frac{N_{i}}{N}\ln\;(\frac{N_{i}}{N})$, which is present in the two-component fluid, but not in the one-component fluid (27, 28).

In contrast, at low temperatures where the crystal is the stable phase, we find that $\Delta \mu_{\text{2c}}<\Delta \mu_{\text{1c}}$. This is most clearly evident for the systems with the widest patch half-angle ($\theta =26^{{}^{\circ}}$), where there is very little driving force for crystallization in the one-component system. This is because the one-component system with wide patches is able to form a fully connected liquid phase, whose stability competes with that of the crystal phase (11–13). Above a critical *θ*, the fully connected liquid becomes the thermodynamically favored phase at low temperatures due to a configurational entropy contribution to the free energy, thereby preventing crystallization into diamond (13). However, even for patch half-angles narrower than this critical value, crystallization is not observed, as we have shown in Fig. 3 *A–C*. Additionally, the highly associative nature of these particles leads to a significant slowdown in dynamics at low temperatures, making the system essentially a guaranteed glass former (12, 15).

In the two-component system, each particle has only N/2 potential bonding partners as compared to *N* – 1 in the one-component system. As a result, the probability of forming a bond in the two-component fluid must be reduced relative to that of the one-component fluid. This is apparent in *SI Appendix*, Fig. S3, where we show that the bonding probability for the one-component system with $\theta =25^{{}^{\circ}}$ is indeed larger than that of the corresponding two-component system prior to crystallization. This explains why there is a greater thermodynamic driving force for crystallization in the two-component system than in the one-component system upon cooling to moderately low temperatures. Therefore, introducing specificity of interactions to program even-member ring selection rules into a system of tetrahedral patchy particles promotes crystallization not only by reducing the frustration experienced by the system, through the removal of kinetic traps, but also by providing an additional thermodynamic driving force.

Additionally, we note that, as we move from the one-component to the two-component system, through the introduction of specific interactions, the coexistence temperature at $P^{*}=0.03$ changes. As shown in Fig. 4*C*, the coexistence temperature shifts to lower and higher values for narrower and wider patches, respectively, with the cross-over occurring around $22^{{}^{\circ}}$. Again, this can be understood by considering how the relative contributions of energy and entropy to the free energy of the respective fluid phases affect the behavior of the system. The two-component system is less prone to bonding, as shown in *SI Appendix*, Fig. S3 and apparent from the energies per particle of the fluid phases at the coexistence temperature (Fig. 4*D*). Fig. 4*E* shows that the coexistence is with a “gas” phase in the case of the one-component system for low *θ* values, but crosses to one with a dense liquid phase for higher *θ* values when we compare the coexistence densities with the corresponding critical densities (29). For the two-component system, the coexistence appears to be always with the “gas” phase. As a result, crystallization occurs via different nucleation mechanisms in the two models, leading to the different *θ* dependence of $T_{\text{coex}}^{*}$, and to the consequent crossing of $T_{\text{coex}}^{*}$ between the two models (Fig. 4*C*). For one-component systems with wider patches, where a network fluid can form, the energy in the liquid phase reaches values similar to the one of the crystal (Fig. 4*D*), and crystal formation becomes essentially controlled by an entropic balance (13).

Our finding that there is an additional thermodynamic driving force for crystallization in the two-component system, involving specificity of interactions, raises the question of whether augmentation of specific interactions would further favor the self-assembly of a diamond crystal. In order to address this question, we considered a two-component system of tetrahedral patchy particles where the four patches are of distinct “colors,” allowing for the formation of bonds only between patches of the same color, provided that the patches belong to particles of different species. This two-component system of “chromatic” patchy particles, which resembles systems that have previously been investigated for the self-assembly of a diamond crystal (6, 30, 31), maintains the same ring selection rules previously imposed to favor crystallization, while increasing the specificity of the interactions. Therefore, we might expect the thermodynamic driving force for crystallization in such a system to be even greater than the two-component system where the patches are not distinguished (30). *SI Appendix*, Fig. S4 shows the results of our Monte Carlo simulations of a two-component (1:1) system of *N* = 1000 chromatic tetrahedral patchy particles in the *NVT* ensemble with $\theta =25^{{}^{\circ}}$. We find that, indeed, only even-member rings form as per our ring selection strategy, but we do not observe crystallization during the course of our simulations, suggesting that this chromatic two-component system is “overspecific,” and particles struggle to identify four neighbors in their immediate vicinity with the right patches available for bonding. This observation also suggests the possibility of a trade-off between increasing the height of the kinetic barriers associated with crystallization and increasing the thermodynamic driving force for crystallization upon introducing specific interactions to a system of tetrahedral patchy particles.

The two main diamond polytypes, cubic and hexagonal diamond, differ only in a single dimension, and can, in fact, be viewed as the limiting cases of a series of diamond polytype structures (32, 33). These different polytype structures can be considered as stacking hybrids of cubic and hexagonal diamond; however, the periodicity of any such polytype can always be described by a hexagonal unit cell (33). Generally, it has been observed that the self-assembly of tetrahedral patchy particles into diamond gives rise to one of these hybrid diamond polytype structures, rather than a pure cubic or hexagonal diamond crystal (11, 12, 34). It is reasonable to anticipate that self-assembled diamond structures will consist of approximately equal proportions of cubic and hexagonal polytypes, due to a marginal free-energy difference between them (11). Similar observations and arguments have been made for the related tetrastack crystals formed from triblock patchy particles (17, 26, 35). However, it was shown that the geometry of the patches influenced the relative stability of cubic and hexagonal tetrastack structures (35), with cubic tetrastack found to become more stable relative to hexagonal tetrastack with increasing patch width, most likely due to the cubic polytype having a more favorable rotational entropy (35, 36).

**Fig. 5.**
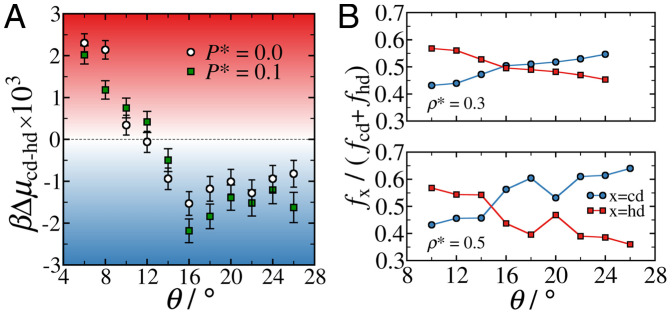
Dependence of diamond polytype selection on the patch width in the two-component system of tetrahedral patchy particles. (*A*) Patch half-angle (*θ*) dependence of the chemical potential difference (Δμ) between the cubic diamond (cd) and hexagonal diamond (hd) crystal structures of a two-component system of *N* = 1800 tetrahedral patchy particles at a temperature of $k_{\text{B}}T/\varepsilon =0.1$ and pressures of $P^{*}=P\sigma^{3}/\varepsilon =0.0,0.1$. The error bars represent the SE in the values, as determined by block averaging. (*B*) Relative fractions of particles in a local cubic diamond ($f_{\text{cd}}$) and hexagonal diamond ($f_{\text{hd}}$) environment following spontaneous crystal formation in systems of *N* = 4000 two-component tetrahedral patchy particles with varying *θ*, at a constant density of $\rho^{*}=N\sigma^{3}/V=0.3$ and $\rho^{*}=0.5$. Each data point corresponds to the average over 25 independent simulations. Note that spontaneous nucleation is observed at different temperatures for different densities and patch half-angles.

The influence of patch geometry on the relative stability of the two diamond polytypes composed of tetrahedral patchy particles, and their relative abundance in self-assembled diamond crystals, has not previously been investigated. This is presumably due to the limited window of patch widths where the spontaneous crystallization of diamond can be observed for a one-component system of tetrahedral patchy particles (9, 11, 14). As we have established, the two-component system under consideration here provides access to the diamond crystals, via self-assembly, for a much wider range of patch half-angles. Fig. 5*A* shows the dependence of the chemical potential difference between the cubic and hexagonal diamond structures ($\Delta \mu_{\text{cd}-\text{hd}}=\mu_{\text{cd}}-\mu_{\text{hd}}$) for the two-component system as a function of the patch half-angle *θ*. As expected, we find that the chemical potential difference is relatively small, and is, in fact, of the same order as the free-energy difference between the face-centered cubic and hexagonal close-packed structures formed by hard spheres (37, 38). We find hexagonal diamond to be marginally more stable for systems with narrow patches ($\theta ≲12^{{}^{\circ}}$), whereas cubic diamond is the more stable polytype for larger *θ* values.

Given the small chemical potential difference, we would not anticipate one polytype to be noticeably favored over the other in self-assembled diamond crystals. To verify whether this is indeed the case, we show, in Fig. 5*B*, the *θ* dependence of the relative fraction of particles in cubic diamond ($f_{\text{cd}}$) and hexagonal diamond ($f_{\text{hd}}$) environments following spontaneous crystallization in two-component systems of *N* = 4000 tetrahedral patchy particles in Monte Carlo simulations. We investigated tetrahedral patchy particles with $\theta =10^{{}^{\circ}},12^{{}^{\circ}},\ldots ,26^{{}^{\circ}}$, where, for each of the nine values of *θ* considered, we performed 25 independent simulations at two different densities: $\rho^{*}=0.3$ and $\rho^{*}=0.5$. We find that hexagonal diamond is more abundant than cubic diamond for systems with narrow patches ($\theta ≲14^{{}^{\circ}}$), while the opposite is true for systems with wider patches. Additionally, the relative proportion of cubic diamond, given by the cubicity, gradually increases (and, hence, the relative proportion of hexagonal diamond gradually decreases) with increasing *θ* at both densities considered. This trend generally agrees with our free-energy calculations; however, we find that $\Delta \mu_{\text{cd}-\text{hd}}$ plateaus around $\theta =16^{{}^{\circ}}$. Therefore, it is likely that there is an additional kinetic driving force whose effect is also dependent on the patch width. This mirrors observations that have been reported regarding the crystallization of ice, where it has been shown that kinetics play a dominant role in determining the relative fraction of cubic and hexagonal ice in large crystallites (39, 40).

In order to determine whether the cubicity in the self-assembled crystalline structures depends on the size of the system, we performed additional sets of 25 independent simulations for two-component systems of *N* = 1000, 8000, and 16,000 designer tetrahedral patchy particles in a 1:1 mixture with $\theta =20^{{}^{\circ}}$ at a density of $\rho^{*}=0.5$. *SI Appendix*, Fig. S5 shows the average cubicity as a function of the system size at $T^{*}=0.155$. It appears that there are some small finite size effects in systems with *N* < 4000 in our simulations, but the cubicity does not noticeably change from a value around 54% when N≥4000. We therefore further compare the average cubicity for two-component systems of *N* = 4000 and 8,000 particles with $\theta =25^{{}^{\circ}}$. We also find that the cubicity, on average, does not practically change, with a value around 65%. These results suggest that the trend in the cubicity (also in the relative fraction of hexagonal diamond) presented in Fig. 5 is not an artifact of finite size effects.

## Conclusions

The self-assembly of the diamond lattice, either cubic, hexagonal, or mixed, from a melt of identical tetrahedral patchy particles, despite being thermodynamically favored, is hampered by the propensity to form disordered arrested states for wide patches (13), and to form five-member ring–rich structures (clathrates) for narrower patches (14). We have established here the conditions to overcome these two bottlenecks and demonstrated a reliable bottom-up route to diamond, simply going from a one-component to a two-component system of tetrahedral patchy particles, where interactions are only allowed between patches on particles of different species.

Our two-component system of designer tetrahedral patchy particles crystallizes into diamond much more readily, as compared to the one-component system, due to two factors: 1) there is no driving force for the formation of odd-member rings, thereby removing kinetic traps from the energy landscape; 2) there is an enhanced thermodynamic driving force for crystallization from the fluid phase due to a decrease in the concentration of potential bonding partners (i.e., particles of another species) for each particle. The removal of kinetic traps from the energy landscape is “programmed” into the design of the two-component system, while the enhanced thermodynamic driving force can be considered as an emergent feature of our design rules, also favoring the formation of diamond. Understanding the mechanism for the promotion of crystallization via the selection of correct ring sizes in the context of colloidal diamond will have general implications for programmed self-assembly of colloidal open crystals. Such mechanistic understanding may also provide insight into the crystallization process at the molecular level in related systems (e.g., tetrahedral network-forming liquids, including water, silica, and silicon).

The specificity in the interactions between patches on different species of tetrahedral patchy particles in the two-component system can be experimentally realized through the use of DNA-mediated interactions (19, 21). The two-component system, therefore, presents itself as a synthetically feasible approach for the self-assembly of a diamond lattice of spheres, which remains a major outstanding problem. Our simulations predict the self-assembly of a mixture of cubic and hexagonal diamond in this route. However, the width of the patches can be used as a handle to control their relative proportions, with wider patches leading to a greater proportion of the cubic polytype. This is likely to be of particular importance in the context of the photonic applications of the self-assembled structures. It was recently shown that randomly stacked diamond structures can display complete photonic band gaps, with those possessing more cubic character generally having superior photonic properties (18).

### Materials and Methods

## Model

We employ the Kern–Frenkel pair potential (22), which has been extensively used to study patchy particles in general and tetrahedral patchy particles in particular (9, 14, 24, 35, 41). Each spherical particle is considered to have a hard core, whose surface is decorated with four circular attractive patches in a tetrahedral symmetry. The effective potential for a pair of patchy particles *v_ij_* is given by

[1] $$v_{ij}(r_{ij},\Omega_{i},\Omega_{j})=v_{ij}^{\text{hs}}(r_{ij})+v_{ij}^{\text{sw}}(r_{ij})\sum_{\alpha ,\beta =1}^{4}f(r_{ij},{\hat{n}}_{i}^{\alpha },{\hat{n}}_{j}^{\beta }),$$

Where $r_{ij}=|r_{ij}|$ is the center-to-center distance between particles *i* and *j*, and $\Omega_{i}$ and $\Omega_{j}$ describe the orientations of particles *i* and *j*, respectively; $v_{ij}^{\text{hs}}$ is the hard-sphere pair potential,

[2] $$v_{ij}^{\text{hs}}(r_{ij})=\{\begin{array}{ll} \infty & \text{if}\;\;r_{ij}<\sigma \\ 0 & \text{otherwise} \end{array},$$

with *σ* representing the hard-sphere diameter; $v_{ij}^{\text{sw}}$ is a square-well potential given by

[3] $$v_{ij}^{\text{sw}}(r_{ij})=\{\begin{array}{ll} -\varepsilon & \text{if}\;\;\sigma \leq r_{ij}\leq (1+\delta )\sigma \\ 0 & \text{otherwise} \end{array},$$

where *ε* is the well depth, and *δ* controls the range of the attraction. Finally, the factor $f(r_{ij},{\hat{n}}_{i}^{\alpha },{\hat{n}}_{j}^{\beta })$ controls the angular dependence of the interaction between two patches, and is given by

[4] $$f(r_{ij},{\hat{n}}_{i}^{\alpha },{\hat{n}}_{j}^{\beta })=\{\begin{array}{ll} 1 & \text{if}\;\;{\hat{n}}_{i}^{\alpha }\cdot {\hat{r}}_{ij}>\cos\;\theta \;\;\text{and}\;\;{\hat{n}}_{j}^{\beta }\cdot {\hat{r}}_{ji}>\cos\;\theta \\ 0 & \text{otherwise} \end{array},$$

where ${\hat{n}}_{i}^{\alpha }$ is a normalized vector from the center of particle *i* in the direction of the center of patch *α* on its surface, and thus depends on $\Omega_{i}$. Similarly, ${\hat{n}}_{j}^{\beta }$ is a normalized vector from the center of particle *j* in the direction of the center of patch *β*, and thus depends on $\Omega_{j}$. The width of the patches is controlled by *θ*, which represents the half-opening angle of the patches. The pairwise additive approximation is used to obtain the total potential energy of the system, V.

For the one-component system, all particles are considered to be identical, and are decorated with patches that are taken to be of equal size, range, and strength. In the two-component system, the two types of particles, labeled A and B, are distinct, despite having the same diameter and attractive patches equal in size and range of interactions. The particles are distinguished as $\varepsilon_{\text{AA}}=\varepsilon_{\text{BB}}=0$ and $\varepsilon_{\text{AB}}=\varepsilon$. That is, there is thermodynamic driving force for bonds only between the two distinct types of particles.

In the present study, we used reduced units: the length in units of *σ*, the energy in units of *ε*, and the temperature in units of $\varepsilon /k_{\text{B}}$, with the Boltzmann constant $k_{\text{B}}$ taken to be equal to one.

### Monte Carlo Simulations

All Monte Carlo simulations, unless otherwise stated, were carried out with systems contained in a cubic box under periodic boundary conditions, using the minimum image convention. Each spherical particle with decorated patches is treated as a rigid body, whose orientational degrees of freedom were represented by quaternions. The potential energy was calculated using a spherical cutoff of 1.2σ, and a cell list was used for efficiency (42). For *NVT* Monte Carlo simulations, each Monte Carlo cycle consisted of *N* translational or rotational single-particle moves, chosen at random with equal probabilities. Additionally, during each cycle of an isothermal–isobaric (*NPT*) Monte Carlo simulation, a volume move was attempted with a probability of 1/N (42).

### Crystal Structure Characterization

In order to assign particles to local environments of cubic diamond, hexagonal diamond, or clathrate structures, we used the translational order correlation parameter $d_{l}(i,j)$, which is based on the Steinhardt bond-orientational order parameters (43, 44),

[5] $$q_{lm}(i)=\frac{1}{N_{\text{B}}(i)}\sum_{j=1}^{N_{\text{B}}(i)}Y_{lm}(r_{ij}),$$

where $N_{\text{B}}(i)$ is the number of first nearest neighbors (a particle shares a patch–patch bond with its nearest neighbors) of particle *i*, $r_{ij}$ is the vector connecting the centers of particles *i* and *j*, and $Y_{lm}$ is the spherical harmonic with total angular momentum *l* and projection −l≤m≤l. The translational order correlation parameter can then be defined as (12, 26, 45–47)

[6] $$d_{l}(i,j)=\text{Re}(\frac{q_{l}(i)\cdot q_{l}^{*}(j)}{\left|q_{l}(i)||q_{l}(j)\right|}),$$

where $q_{l}(i)\cdot q_{l}^{*}(j)=\sum_{m=-l}^{l}q_{lm}(i)\cdot q_{lm}^{*}(j)$, and * indicates the complex conjugate.

In this analysis, $d_{l}(i,j)$ is used to determine whether the local environments of particles *i* and *j* are sufficiently similar, and hence share a crystalline bond. Those particles, which possess a certain threshold number of crystalline bonds, are labeled as crystalline. Therefore, $d_{l}(i,j)$ can be used to directly count the number of solid particles in a system (45). Here, we followed a protocol similar to what was laid out in ref. 12: Particles in a crystalline environment were identified using $d_{3}(i,j)$, where two particles were considered to share a staggered bond if $-1\leq d_{3}(i,j)\leq -0.85$ and an eclipsed bond if $-0.3\leq d_{3}(i,j)\leq 0.1$. Particles with exactly four bonds were taken to be crystalline, and their local environments were further characterized as cubic diamond (four staggered bonds), hexagonal diamond (three staggered bonds plus one eclipsed bond), or clathrate (four eclipsed bonds), depending upon the number of staggered and eclipsed bonds each particle possessed.

### Ring Statistics

We calculated the number of rings ($N_{{\text{R}}_{l}}$) with sizes l∈[4−8], in a system of *N* particles using a graph theoretical approach. In this approach, a configuration is represented by a periodic undirected graph G, where the vertices ($V_{G}$) of the graph denote the particle centers, and the edges ($E_{G}$) denote the bonds between the particles. Two particles are considered “bonded” if there is an attractive interaction between patches on them, and we define a ring as a closed path in this graph (i.e., a path whose first and final vertices are the same). For each vertex $V_{G}^{i}$ in the graph, we determine all distinct paths using a depth-first search (DFS) traversal, up to the chosen maximum *l*, making sure to prune paths with edges between nonadjacent vertices along the path to ensure any potential ring does not contain any internal edges. Then, rings are identified as paths where the first and last vertices are the same. In order to avoid overcounting the number of rings in the system, following the extraction of all relevant paths starting from vertex $V_{G}^{i}$ (and hence rings containing $V_{G}^{i}$), all edges connected to that vertex are removed from the graph prior to initiating the DFS traversal from the next vertex. We also take into consideration the double counting of a ring, which results from the undirected nature of the graph.

### Free-Energy Calculations

We used thermodynamic integration to determine the chemical potential of the different phases formed by the one- and two-component patchy particle systems as a function of temperature along an isobar. The working expression to perform these calculations is (42, 48)

[7] $$\beta_{2}\mu (N,P,T_{2})=\beta_{1}\mu_{\text{ref}}(N,P,T_{1})-\int_{T_{1}}^{T_{2}}\text{d}T{\langle \frac{H(N,P,T)}{Nk_{\text{B}}T^{2}}\rangle }_{T},$$

where $\beta ={\left(k_{\text{B}}T\right)}^{-1},\;H=V+PV$ is the enthalpy, and $\mu_{\text{ref}}$ is a reference chemical potential. We performed a series of *NPT* simulations at the chosen pressure to determine the enthalpy at discrete temperature intervals; the integrand in Eq. **7** was then evaluated numerically using a cubic spline interpolation. Reference chemical potentials were determined from reference free energies and the equation of state using the relation $\beta \mu_{\text{ref}}=\beta (f_{\text{ref}}+PV/N)$, where $f_{\text{ref}}$ is a reference free energy per particle at the chosen thermodynamic state (42, 48).

The reference free energies of the fluid phase were determined by thermodynamic integration along an isochore, using the hard-sphere fluid as the reference at *β* = 0 (42, 49),

[8] $$\beta f_{\text{ref}}(N,V,T)=\beta f_{\text{hs}}(N,V)+\int_{0}^{\beta }\text{d}\beta^{\prime }{\langle \frac{V(N,V,\beta^{\prime })}{N}\rangle }_{\beta^{\prime }},$$

where $f_{\text{hs}}$ is the free energy per particle for the hard-sphere fluid, which was evaluated using the Carnahan–Starling equation of state (50). For the two-component fluid, it is necessary to also include the entropy of mixing in this expression (27, 28). As before, we carried out a series of *NVT* simulations to determine the potential energy at discrete temperature intervals, and the integrand in Eq. **8** was evaluated numerically.

The reference free energies of the cubic and hexagonal diamond crystals were calculated using the Einstein crystal method (24, 42, 48, 51). We note that there is an extra configurational entropy contribution to the free energy of the two-component crystalline phases that accounts for the fact that two degenerate structures exist for each crystal, where the particle identities are swapped: $S_{\text{conf}}/(Nk_{\text{B}})=\ln\;(2)/N$. When comparing the chemical potentials of the two diamond polytypes, Monte Carlo simulations were performed in an orthorhombic cell commensurate with both structures. The edge lengths of the orthorhombic cell, during *NPT* simulations, were allowed to fluctuate independently.

## Supplementary Material

Supplementary File

## Data Availability

Datasets and software have been deposited in the University of Birmingham edata Repository and can be accessed from https://edata.bham.ac.uk/711/.
