# Supplementary material for: Facile self-assembly of colloidal diamond from tetrahedral patchy particles via ring selection
Source: Proc Natl Acad Sci U S A. 2021 Nov 24;118(48):e2109776118. doi: 10.1073/pnas.2109776118 (PMC8640719; doi:10.1073/pnas.2109776118)
Supplement: Supplementary File [file pnas.2109776118.sapp.pdf]

PNAS
www.pnas.org

Supplementary Information for

Facile self-assembly of colloidal diamond from tetrahedral patchy particles via ring selection

Andreas Neophytou[a], Dwaipayan Chakrabarti[a], Francesco Sciortino[b]

[a]School of Chemistry, University of Birmingham, Edgbaston, Birmingham B15 2TT, United Kingdom

[b]Dipartimento di Fisica, Sapienza Università di Roma, Piazzale Aldo Moro 5, 00185 Roma, Italy

[1]E-mail: d.chakrabarti@bham.ac.uk
[2]E-mail: francesco.sciortino@uniroma1.it

This PDF file includes:

Supplementary text
Figs. S1 to S5
SI References

Supporting Information Text

Monte Carlo Simulation Details

One- and two-component systems with narrow patches. We performed a series of Monte Carlo simulations for one- and two-component systems of $N = 1000$ tetrahedral patchy particles with patch half-angle $\theta = 12^\circ$ and at a density of $\rho^* = N\sigma^3/V = 0.3$. Each system was gradually cooled to a temperature of $T^* = k_B T/\varepsilon = 0.1$, starting from an equilibrium configuration obtained by melting a simple cubic lattice at $T^* = 1$. For the one-component system, the following cooling protocol was followed in steps: $T^* = 1.0 \to 0.2$ with $\Delta T^* = -0.2$, $T^* = 0.18 \to 0.14$ with $\Delta T^* = -0.02$, $T^* = 0.135 \to 0.13$ with $\Delta T^* = -0.005$, and $T^* = 0.12 \to 0.1$ with $\Delta T^* = -0.01$. At each temperature between $T^* = 1.0$ and $T^* = 0.16$, 1 million Monte Carlo cycles were run, then 3 million Monte Carlo cycles were run at $T^* = 0.14$ and 30 million Monte Carlo cycles were run at $T^* = 0.135$, before running 2 million Monte Carlo cycles at each temperature between $T^* = 0.13$ and $T^* = 0.1$. For the two-component system, we followed the following cooling protocol in steps: $T^* = 1.0 \to 0.2$ with $\Delta T^* = -0.2$, $T^* = 0.18 \to 0.14$ with $\Delta T^* = -0.02$, $T^* = 0.135 \to 0.13$ with $\Delta T^* = -0.005$, and then at $T^* = 0.128, 0.125, 0.12, 0.1$ in the sequence. We carried out 1 million Monte Carlo cycles at each temperature between $T^* = 1.0$ and $T^* = 0.14$, 3 million Monte Carlo cycles at $T^* = 0.135$, 10.5 million Monte Carlo cycles at $T^* = 0.13$ and $T^* = 0.128$, and 2 million Monte Carlo cycles at each temperature between $T^* = 0.125$ and $T^* = 0.1$.

Additionally, we performed a series of Monte Carlo simulations for the one-component system of $N = 1000$ tetrahedral patchy particles with $\theta = 12^\circ$ at $\rho^* = 0.5$, starting from an equilibrium configuration at $T^* = 1$. We followed the following cooling protocol in steps: $T^* = 1.0 \to 0.2$ with $\Delta T^* = -0.2$, $T^* = 0.18 \to 0.16$ with $\Delta T^* = -0.02$, $T^* = 0.15 \to 0.145$ with $\Delta T^* = -0.005$, and then at $T^* = 0.144$, before lowering the temperature further: $T^* = 0.14 \to 0.1$ with $\Delta T^* = -0.01$. We carried out 1 million Monte Carlo cycles at each temperature between $T^* = 1.0$ and $T^* = 0.16$, 10 million Monte Carlo cycles run at each temperature between $T^* = 0.15$ and $T^* = 0.144$, and then 3 million Monte Carlo cycles at each temperature between $T^* = 0.14$ and $T^* = 0.1$.

One- and two-component systems with wider patches. We performed a series of Monte Carlo simulations for one- and two-component systems of $N = 1000$ tetrahedral patchy particles with patch half-angle $\theta = 25^\circ$ and at a density of $\rho^* = 0.5$. Each system was gradually cooled to a temperature of $T^* = 0.1$, starting from an initial simple cubic lattice which was melted at $T^* = 1$. For both systems, the following cooling protocol in steps was followed: $T^* = 1.0 \to 0.6$ with $\Delta T^* = -0.2$, $T^* = 0.5 \to 0.2$ with $\Delta T^* = -0.1$, $T^* = 0.18 \to 0.16$ with $\Delta T^* = -0.02$, $T^* = 0.155 \to 0.15$ with $\Delta T^* = -0.005$, and $T^* = 0.14 \to 0.1$ with $\Delta T^* = -0.01$. We carried out 1 million Monte Carlo cycles at each temperature between $T^* = 1.0$ and $T^* = 0.3$, 5 million Monte Carlo cycles at each temperature between $T^* = 0.2$ and $T^* = 0.16$, 15 million Monte Carlo cycles at $T^* = 0.155$ and 0.15, and 5 million Monte Carlo cycles at each temperature between $T^* = 0.14$ and $T^* = 0.1$.

Two-component systems with a range of patch widths. To investigate the influence of the patch width on the relative proportions of cubic and hexagonal polytypes in the self-assembled diamond crystals, we performed a series of Monte Carlo simulations of two-component systems of $N = 4000$ designer tetrahedral patchy particles with equal-size patches at densities of $\rho^* = 0.3$ and $\rho^* = 0.5$. For these systems, the half-opening angles for the patches were varied over a range as follows: $\theta = 10^\circ, 12^\circ, 14^\circ, 16^\circ, 18^\circ, 20^\circ, 22^\circ, 24^\circ, 26^\circ$. For each of these nine half-opening angles, 25 independent Monte Carlo simulations were performed, by subjecting the system to one-step quenching to investigate spontaneous crystallization. The starting configurations were obtained by melting an initial face-centered cubic lattice at $T^* = 1$. At $\rho^* = 0.3$, each system was cooled to the following temperatures: $T^* = 0.115, 0.125, 0.130, 0.14, 0.145, 0.145, 0.145, 0.145, 0.145$ (given in the ascending order of θ). At $\rho^* = 0.5$, each system was cooled to the following temperatures: $T^* = 0.125, 0.135, 0.143, 0.15, 0.15, 0.155, 0.155, 0.155, 0.15$ (given again in the ascending order of θ). For each density and patch half-angle, a set of initial simulations was performed at different temperatures, carrying out 5 million Monte Carlo cycles. The temperatures given above correspond to the highest temperature where spontaneous crystallization was observed in at least one of those simulations. The simulations at each of those temperatures were then continued until all 25 runs had crystallized, allowing for the same number of Monte Carlo cycles for a given patch width at a particular density.

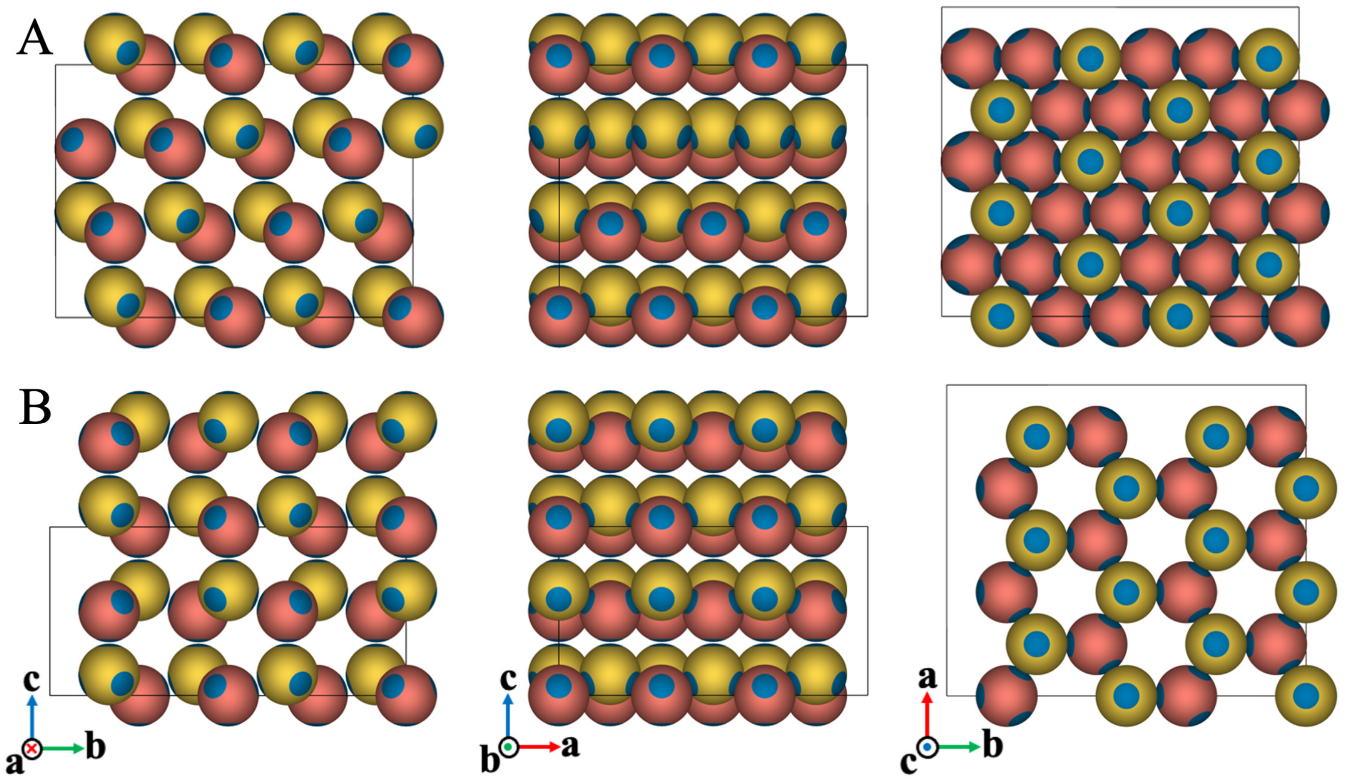

Fig. S1. Cubic and hexagonal diamond crystal structures formed by a two-component (1:1) system of tetrahedral patchy particles. Orthorhombic supercells of the (*A*) cubic and (*B*) hexagonal diamond polytypes in different views along directions orthogonal to high symmetry faces.

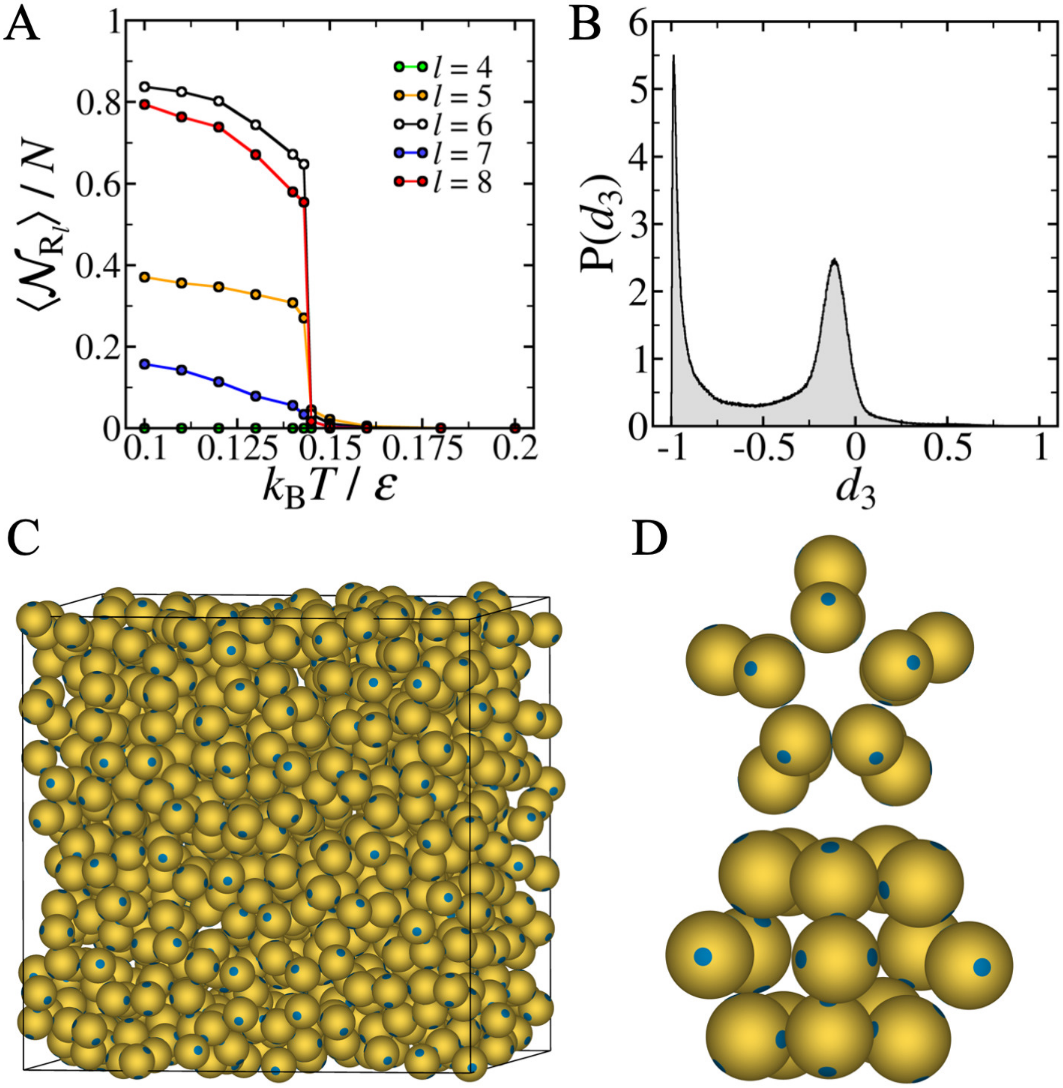

Fig. S2. Self-assembly of an amorphous clathrate phase in a one-component system of $N = 1000$ tetrahedral patchy particles with patch half-angle $\theta = 12^{\circ}$ at a density of $\rho^* = N\sigma^3/V = 0.5$. (*A*) Evolution of the average number of rings of size l ($\mathcal{N}_{R_l}$) with temperature. (*B*) Probability distribution function of the translational-order correlation parameter d_3 (defined in the methods section) at a temperature of $k_B T/\varepsilon = 0.1$. (*C*) Snapshot of a representative configuration of the system at a temperature of $k_B T/\varepsilon = 0.1$. (*D*) Representative views of a $5^2 6^5$ cage, characteristic of the amorphous clathrates (1).

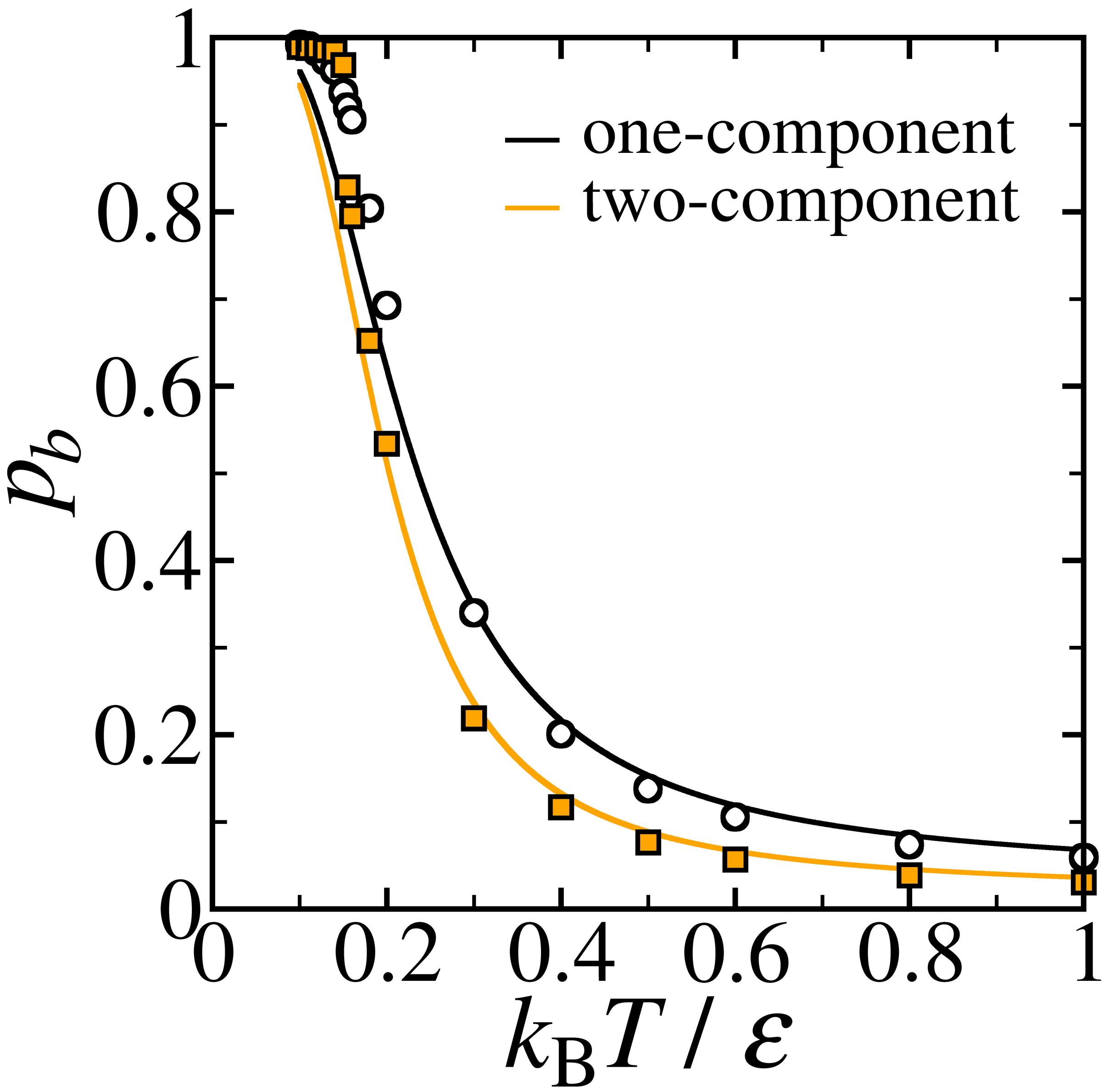

Fig. S3. Bonding probabilities for one- and two-component systems of tetrahedral patchy particles with patch half-angle $\theta = 25^\circ$ at a density of $\rho^* = N\sigma^3/V = 0.5$ as a function of temperature. The data points correspond to bonding probabilities determined from Monte Carlo simulations of the systems of $N = 1000$ tetrahedral patchy particles and the lines correspond to bonding probabilities determined using the mass action laws (2, 3).

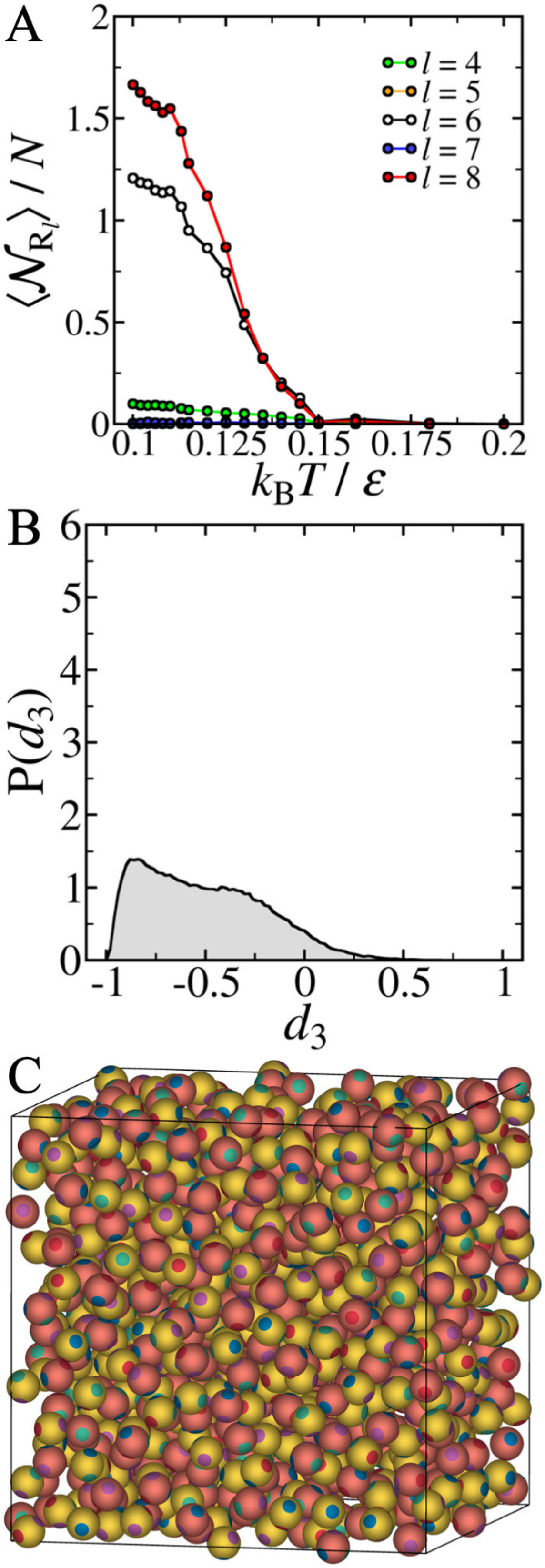

Fig. S4. Self-assembly in a two-component systems of $N = 1000$ chromatic tetrahedral patchy particles with patch half-angle $\theta = 25^{\circ}$ at $\rho^* = N\sigma^3/V = 0.5$. (*A*) Evolution of the average number of rings of length l ($\mathcal{N}_{\mathrm{R}_l}$) with temperature. (*B*) Probability distribution function of the translational-order correlation parameter d_3, defined in the methods section, at a temperature of $k_{\mathrm{B}}T/\varepsilon = 0.1$. (*C*) Representative snapshot of the resulting amorphous network at a temperature of $k_{\mathrm{B}}T/\varepsilon = 0.1$.

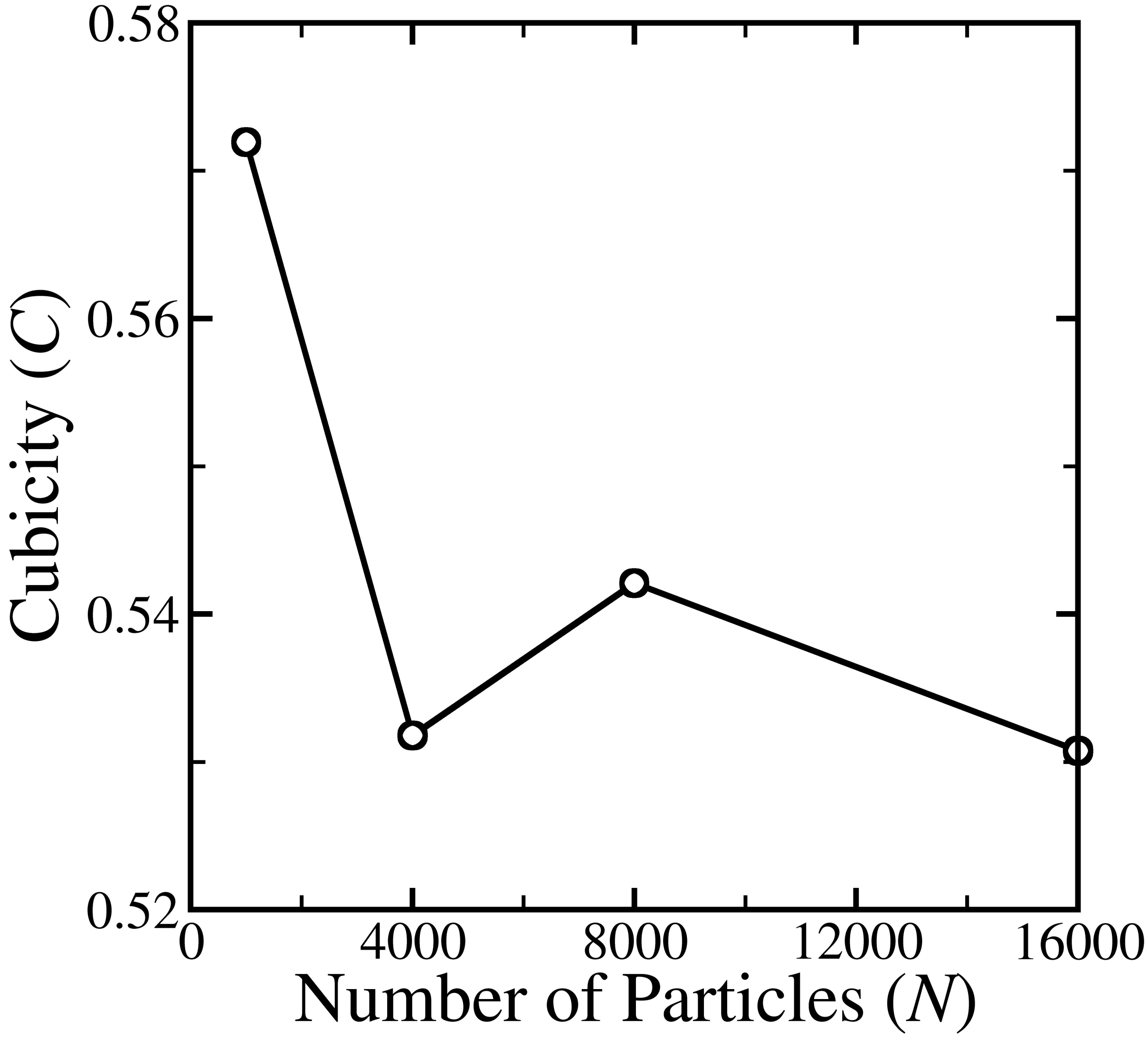

Fig. S5. System-size dependence of the average cubicity, C, in the colloidal diamond crystals self-assembled from two-component (1:1) systems of tetrahedral patchy particles with patch half-angles of $\theta = 20^\circ$ at a density of $\rho^* = 0.5$ and temperature $T^* = 0.155$. For each system size, 25 independent simulations were performed following a one-step cooling from $T^* = 1.0$ and spontaneous nucleation was observed in the course of each simulation.

References

1. EG Noya, I Zubieta, DJ Pine, F Sciortino, Assembly of clathrates from tetrahedral patchy colloids with narrow patches. *J. Chem. Phys.* **151**, 094502 (2019).
2. D de Las Heras, JM Tavares, MM Telo da Gama, Phase diagrams of binary mixtures of patchy colloids with distinct numbers of patches: the network fluid regime. *Soft Matter* **7**, 5615–5626 (2011).
3. D de Las Heras, JM Tavares, MM Telo da Gama, Phase diagrams of binary mixtures of patchy colloids with distinct numbers and types of patches: The empty fluid regime. *J. Chem. Phys.* **134**, 104904 (2011).
